# Correlations between axial stiffness and microstructure of a species of bamboo

**DOI:** 10.1098/rsos.160412

**Published:** 2017-01-18

**Authors:** Sayyad Mannan, J. Paul Knox, Sumit Basu

**Affiliations:** 1 Department of Mechanical Engineering, Indian Institute of Technology Kanpur, Kanpur, Uttar Pradesh 208016, India; 2 Centre for Plant Sciences, Faculty of Biological Sciences, University of Leeds, Leeds LS2 9JT, UK

**Keywords:** bamboo, multiscale modelling, functionally graded material

## Abstract

Bamboo is a ubiquitous monocotyledonous flowering plant and is a member of the true grass family *Poaceae*. In many parts of the world, it is widely used as a structural material especially in scaffolding and buildings. In spite of its wide use, there is no accepted methodology for standardizing a species of bamboo for a particular structural purpose. The task of developing structure–property correlations is complicated by the fact that bamboo is a hierarchical material whose structure at the nanoscopic level is not very well explored. However, we show that as far as stiffness is concerned, it is possible to obtain reliable estimates of important structural properties like the axial modulus from the knowledge of certain key elements of the microstructure. Stiffness of bamboo depends most sensitively on the size and arrangement of the fibre sheaths surrounding the vascular bundles and the arrangement of crystalline cellulose microfibrils in their secondary cell walls. For the species of bamboo studied in this work, we have quantitatively determined the radial gradation that the arrangement of fibres renders to the structure. The arrangement of the fibres gives bamboo a radially graded property variation across its cross section.

## 1. Introduction

Bamboo, a member of the true grass family *Poaceae*, is a ubiquitous monocotyledonous flowering plant, of which there are over 75 genera and 1250 species [1]. Out of about 130 species of wild and cultivated bamboo occurring in India, 13 belonging to seven genera have been recommended by the National Bamboo Mission for use in various traditional and industrial applications (http://nbm.nic.in/Achievement/Handbook%20on%20Bamboo.pdf). A major use of some of these species is in construction and scaffolding. In fact, Bhalla *et al.* [2] have demonstrated that modern structural engineering principles can be used to design robust structures (e.g. a large shed adhering to established design norms for steel and concrete structures) with bamboo as the major structural element.

In their design, Bhalla *et al.* [2] have assumed that bamboo (in particular, the species *Dendrocalamus giganteus*) has a Young's modulus of 14 GPa, and tensile and compressive yield strengths of 120 and 55 MPa, respectively. While these values are impressive for a structural material (especially given that the density is about $700\;\mathrm{kg}\;m^{-3}$ which results in a significantly higher strength to weight ratio compared with, say, steel; see also [3] for detailed comparisons), structure–property correlations for various species of bamboo are not very well developed. With a view to determine the mechanical stiffness and strength of bamboo, the recent works by Habibi *et al.* [4] and Dixon & Gibson [5] are noteworthy. For specific species of bamboo (*Phyllostachys edulis* in the former and *Phyllostachys pubescens* or Moso bamboo in the latter), the stiffness has been obtained quantitatively with due consideration to the distinctive microstructure of bamboo. In particular, as will also be seen from our results, the distribution of the vascular bundles and the fibre bundles around them, play an important role in determining the stiffness. Habibi *et al.* [4] also determine the fracture properties. Comparison between the overall stiffness from flexural tests on a number of different bamboo species has recently been done by Dixon *et al.* [6].

Detailed tests to determine mechanical properties of bamboo stem, which is known as culm, have been reported [7, 8]. Armandei *et al.* [9] have used frequency spectrum analysis on bamboo slices in bending mode to study the variation of its modulus of elasticity. However, the essential features of the bamboo microstructure that contribute to and determine its stiffness have not been clearly identified.

In this paper, we address this issue and attempt to identify the most important microstructural attributes that determine the mechanical stiffness of a bamboo culm, particularly in the axial direction. Although Liese [1] mentions that, compared to the large structural heterogeneity of the 20 000 timber species, the variations among the species of bamboo appear small, we believe that this work will help in identifying the species most suited to a particular structural need.

We show that in order to obtain a reliable estimate of the axial mechanical properties of a particular species of bamboo, knowledge of a small set of key parameters is sufficient. These include the areal distribution of fibres, average orientation angle of the cellulose microfibrils in the secondary cell walls of fibres and size distribution of the fibres. Apparent sweeping simplifications like ignoring the amorphous nature of a part of the cellulose, assuming approximate values of the stiffness of the amorphous non-cellulosic polysaccharides in the microfibrils, size distribution of the parenchyma cells, etc., do not seem to affect the stiffness estimate significantly.

## 2. Bamboo: a multiscale, hierarchical graded composite

### 2.1. Overall structure of bamboo culm

**Figure 1.**
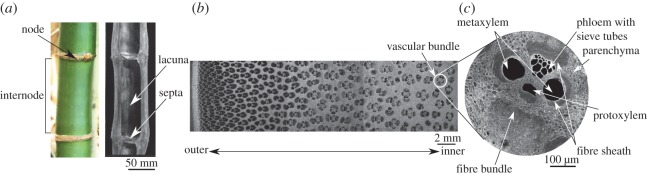
Hierarchical microstructure of bamboo.

The bamboo culm is approximately cylindrical, thick walled shell (the cavity is called lacuna) with periodic transverse partitions called septa (figure 1*a*), whose purpose seems to be to prevent ovalization of the cross section during bending, thereby preventing Brazier buckling [10]. The cross section of culm, shown in figure 1*b*, has non-uniformly distributed vascular bundles, with higher areal concentration being close to the outer wall. As in the optical micrograph shown in figure 1*c*, the vascular bundles are easily distinguished by their dark sclerenchymatous cells against the lighter ground parenchyma. The vascular bundles are generally composed of two metaxylem vessels, phloem, protoxylem attached fibre sheaths and fibre bundles [1].

Appearance of the vascular bundles in a transverse section of bamboo varies between species. Grosser & Liese [11, 12] have identified four different types of vascular bundles in bamboo, distinguished by the number of vascular strands and the arrangement of supporting sclerenchyma cells. Although the type varies between basal and middle parts, as well as within a cross section, the species of bamboo studied here consists primarily of type IV vascular bundles. A typical example of a vascular bundle is shown in figure 1*b*. The vascular bundle contains the phloem with small, thin-walled sieve tubes and the metaxylem vessels.

**Figure 2.**
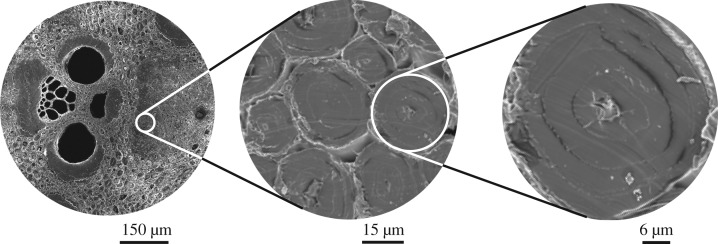
Hierarchical structure of a bamboo fibre.

From the point of view of stiffness, the most important elements of the vascular bundle are the fibres [13, 14], which form around 40% of the sclerenchymatous tissue volume in the culm. Dark areas in figure 1*b* are fibre bundles that surround the conducting elements forming strengthening sheaths. These fibre bundles are composed of close-packed individual fibres (figure 2). Typical length scales of the various elements in the vascular bundle have been shown in figures 1 and 2.

**Figure 3.**
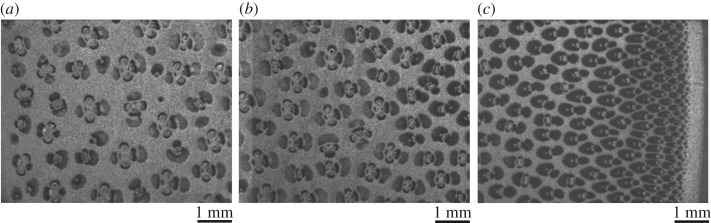
Cross-sectional micrographs show the gradation in the areal density of fibres in (*a*) inner, (*b*) middle and (*c*) outer parts of the cross section.

As mentioned earlier, the structure and density of the vascular bundles change continuously from the inner edge to the periphery. Nearer to the periphery the bundles are smaller and denser. The structure of the fibres differs among the sheaths and from the inner edge to the periphery. Micrographs, showing the distributions of fibre bundles at the inner edge, mid-thickness and the periphery, are shown in figure 3. It should be noted that the fibres have an aspect ratio of 70–150.

**Figure 4.**
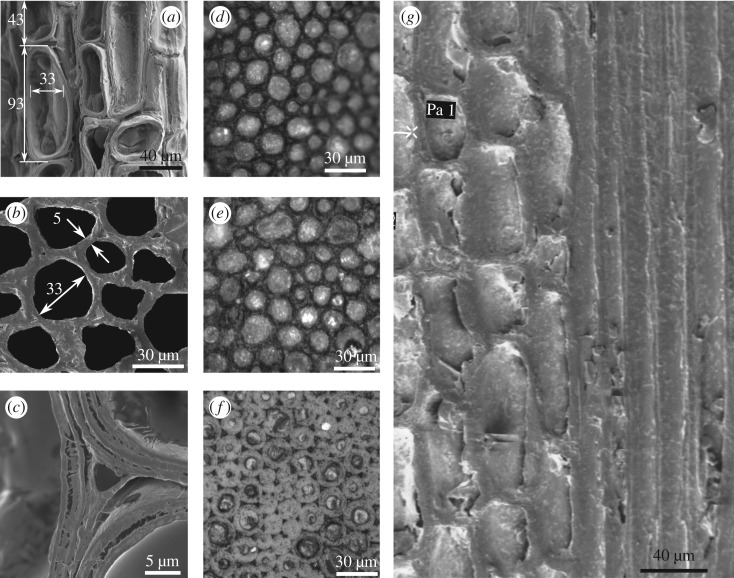
(*a*) Longitudinal and (*b*) cross-sectional micrographs show typical dimensions of parenchyma cells, cell walls in the species *Dendrocalamus strictus*. The wall layers are clearly seen in (*c*). Images in (*d*–*f*) show the fibre sizes at inner, middle and outer locations, respectively. Longitudinal section in (*g*) has parenchyma with the embedded fibres resemble the arrangement of high aspect ratio hard phase in a soft matrix seen in many biological materials [15].

The ground tissue surrounding the vascular bundles is made up of parenchyma, whose percentage varies considerably within the cross section from almost 77% at the inner edge to only 36% at the periphery. Typical dimensions of the key features of these cells shown in figure 4 make it clear that the ground tissue resembles a soft open-cell foam with the vascular bundles, particularly the fibre sheaths, acting as reinforcements. Thus, bamboo is an apt example of a transversely isotropic, functionally graded composite material.

We note in passing that the longitudinal section shown in figure 4*a*,*g* showing the equiaxed parenchyma cells and the long fibres running through illustrates an important point about the microstructure of bamboo internodes. At the most elementary structural level, the microstructure is similar to the generic microstructure of biocomposites such as nacre and collagen [15, 16] and abalone nacre-inspired multilayered materials [17]. The generic microstructure consists of staggered stiffer elements (fibres in the case of bamboo, mineral platelets in collagen and many other bioinspired brick and mortar composites) with large aspect ratios in a soft matrix. It has been suggested [15] that the high stiffness of the structure is achieved by the high aspect ratio of the fibres while the soft parenchymatous matrix helps in load transfer between the fibres through shear. In another study on bioinspired brick and mortar composites, Wilbrink *et al.* [18] have provided scaling relationships between constituent properties and the uniaxial tensile response of synthetic brick and mortar composite materials inspired by nacre. All these models are helpful to understand the toughening mechanisms in many natural biocomposites like bamboo.

### 2.2. Finer structure of bamboo fibres

**Figure 5.**
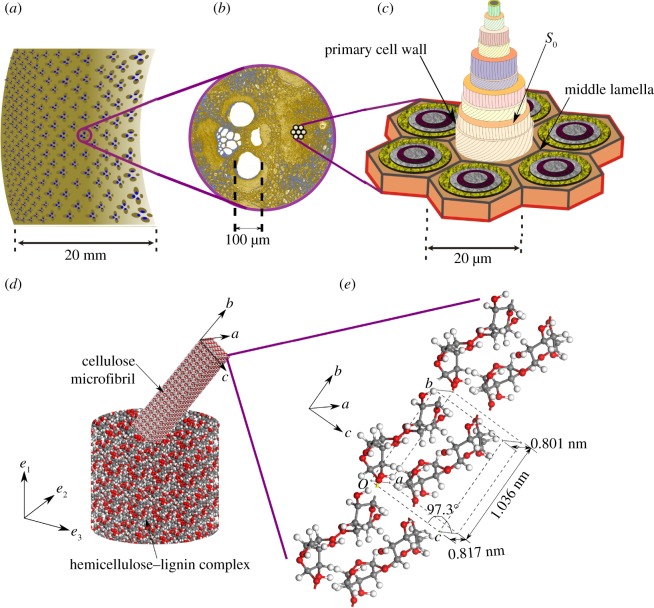
Anenlarged view of typical vascular bundle from a typical cross section in (*a*) is shown in (*b*). Orientation of cellulose microfibrils to the axial direction (according to Parameswaran & Liese [24]) in different lamellae of a fibre cell is shown in (*c*). An individual lamella in (*c*) is, in turn, a composite of largely single crystalline cellulose microfibrils in a lignin–hemicellulose complex (*d*). The reinforcing microfibrils are composed of cellulose chains with unit cell and orientation indicated in (*e*).

As noted earlier, the remarkable mechanical properties of bamboo are attributed to the closely packed fibres constituting the sheaths in the vascular bundles. At the microscopic level, detailed investigations by e.g. Tono & Ono [19] and Parameswaran & Liese [20] have shown that the walls of the fibres are composed of a large number of alternate thin and thick layers. The number of layers varies during the life of the plant [21] but mature fibres generally have 8–10 lamellae. Each layer has cellulose microfibrils oriented at various angles to the fibre axis surrounded by matrix of hemicellulose and lignin [22, 23]. The structure of the multilamellar secondary cell wall of a fibre has been presented by Parameswaran & Liese [24]. The structure is shown in figure 5. Starting from the middle lamella, the primary cell wall and a transition layer $S_{0}$, the microfibril angles alternate between values close to $0^{\circ }$ in the thick lamella and $90^{\circ }$ in the thin ones.

Like other biological materials such as wood, bone, nacre and deep-sea sponges, the hierarchical structure of bamboo extends all the way down to the nanoscale. However, our understanding of the detailed nanoscale structure of bamboo is incomplete. It is known that the major constituent of the lamella is cellulose formed by assembling glucose molecules into microfibrils that have either rectangular or hexagonal cross sections with approximate diameter of 3–5 nm [22]. If the hydrogen bonds between the hydroxyl groups in cellulose form in a particular order, crystalline regions result,^1^ while random hydrogen bonds lead to amorphous regions.

Recent molecular simulations have thrown some light on the structure of lignin and non-cellulosic polysaccharide complex surrounding the cellulose microfibrils and the mechanisms by which they bind to each other. The task is complicated by the fact that the phenolic macromolecule lignin has a large number of possible structures as it is naturally synthesized from the sub-units *p*-hydroxyphenyl, guaiacyl and syringyl following a large number of possible pathways [25]. The non-cellulosic polysaccharides or hemicelluloses act as storehouses of metabolizable energy and have also been shown to be involved in growth regulation and in signalling pathways associated with loss of cell wall integrity [26]. Habibi *et al.* [27] have also done steered molecular dynamics (MD) simulations of the cellulose surrounded by the hemicellulose–lignin complex in bamboo in an effort to better understand its viscoelastic properties.

Monoclonal antibodies coupled with fluorescent moieties, directed against cell wall polysaccharides, can specifically recognize epitopes that form small regions of the polysaccharide molecule. Thin sections of bamboo (about 1 μm in thickness), fixed and treated with a suitable antibody [28], when examined under a fluorescence microscope yield valuable information on the presence or absence of a particular polysaccharide.

**Figure 6.**
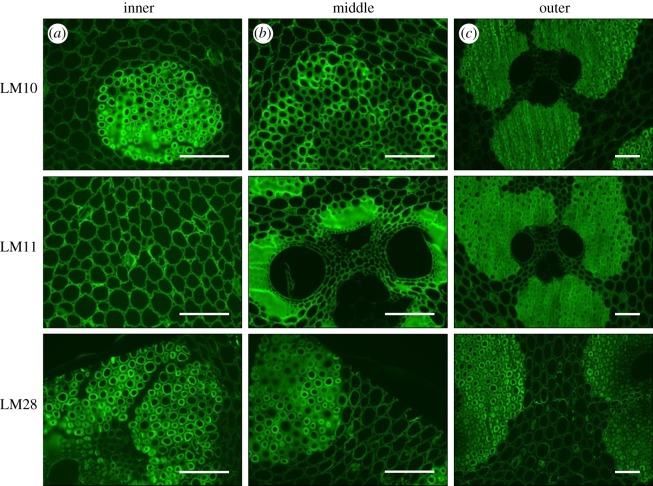
Immunofluorescence images of culm transverse sections labelled with xylan monoclonal antibodies LM10, LM11 and LM28 show strong detection of xylan in secondary cell walls of parenchyma and fibres. Scale bar, 100 μm.

**Figure 7.**
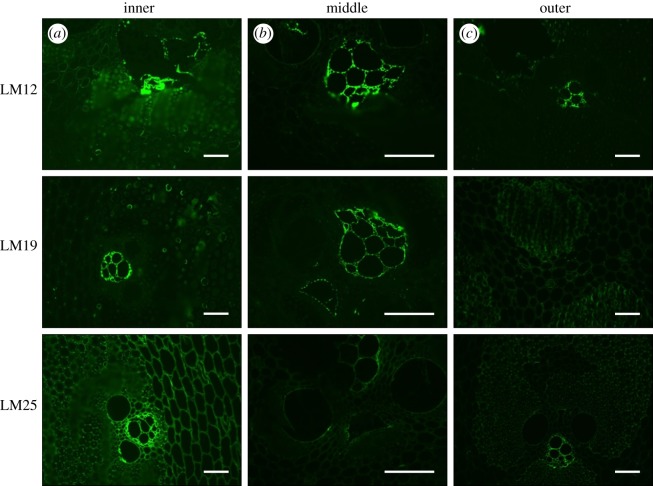
Immunofluorescence images of culm transverse sections labelled with xylan monoclonal antibodies LM12, LM19 and LM25 show detection of xylan close to the phloem walls only. Scale bar, 100 μm.

Figure 6*a–c* shows immunofluorescence images of transverse sections of bamboo labelled with antibodies to LM10 and LM11 xylan epitopes [29] and LM28 to a glucuronoxylan epitope [30]. Note that xylans are a group of hemicelluloses that are abundantly found in plant cell walls. All three antibodies detect xylan in inner, middle and outer parts, respectively, and show strong binding to cell walls of fibre cells. This indicates that the fibres have an abundance of different forms of xylans. On the other hand, probe molecules LM12, LM19 and LM25 [31] which are antibodies to feruloylated xylan, pectic homogalacturonan and xyloglucan, respectively, could detect these molecules close to the phloem walls only as shown in figure 7*a–c*.

As far as the fibres are concerned, these experiments seem to reveal an almost complete picture of the secondary cell wall structure. The thick secondary cell walls are lamellar with cellulose microfibrils oriented at alternately low and high angles to the axis. The cellulose microfibrils are surrounded by the lignin–hemicellulose complex in which the lignin molecules are cross-linked by xylan, arabinoxylan and glucuronoxylan. A schematic summarizing the structure of the fibre cell wall is included in figure 5.

It is believed that the b axis of the cellulose unit cell (shown in figure 5) is oriented along the axis of the microfibrils [32, 33]. In X-ray diffraction (XRD), the strongest reflection comes from (002) planes for $2\theta =22.6^{\circ }$. We also determined the crystallinity index (CI) or degree of crystallinity of cellulose in bamboo fibres to be approximately 84% empirically as CI $=(I_{\max}-I_{\min})/I_{\max}$, where $I_{\max}$ is the height of the peak at $2\theta =22.6^{\circ }$, which represents both crystalline and amorphous cellulose; and $I_{\min}$ is the height of the minimum at $2\theta =16.3^{\circ }$, which represents the amorphous cellulose [34].

Binding between both crystalline and amorphous cellulose and the lignin–hemicellulose complex in bamboo fibre cell wall has recently been studied through MD simulations [22]. The simulations show that, owing to a higher van der Waals interaction, compared with any of the hemicelluloses, lignin adheres strongly to crystalline cellulose. However, the weakest links in the cellulose–lignin–hemicellulose network that makes up the secondary cell walls of the fibres are the interfaces between amorphous cellulose and the lignin–hemicellulose complex.

## 3. Experimental details, materials and methods

A bamboo culm of local variety (species Dendrocalamus strictus) was obtained from the botanical nursery, IIT Kanpur and aged in the open for eight weeks for natural seasoning. The sixth internode from bottom end of the culm (outer diameter 71.8, inner diameter 32.4 and length 270 mm) was selected for all experimental studies reported herein.

**Figure 8.**
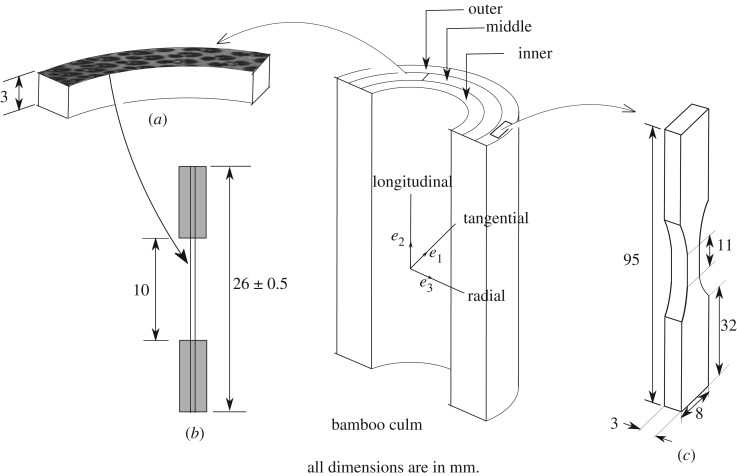
A bamboo culm shows the different test samples that are extracted from it.

Basically, three kinds of samples were prepared from the internode. The cross section of the internode was divided approximately into three regions: inner, middle and outer. Semi-circular transverse slices of 3 mm thickness were made from each region and polished with alumina for further use as shown in figure 8*a*. These were used mainly for electron microscopic studies.

As shown in figure 8*b*, following ASTM recommendations [35], tensile specimens were prepared with the length in the longitudinal direction and properties along the thickness representing those of the inner, middle and outer regions of the transverse cross section.

The third set of specimens (shown in figure 8*c*) was prepared to test the mechanical stiffness of the sclerenchymatous fibres. To this end, the semicircular slices were immersed in 10% NaOH solution for 24 h at room temperature to weaken the bonding between fibre bundles and the parenchymatous cells. It has been shown [8] that alkali treatment degrades the properties of the fibres and the concentration of the NaOH solution and the soaking time needs to be carefully adjusted, so that the matrix only is dissolved. Once the slices are taken out from NaOH solution and washed thoroughly with deionized water, the fibre bundles can be separated easily under a microscope. The extracted bundles were air-dried for 24 h and then cut into pieces 26±0.5 mm in length, for testing in a microtensile set-up. The ends of dried bundles were bonded with epoxy resin to a frame made up of a hard paper.

Tension tests have been conducted on either a universal testing machine fitted with a 10 kN load cell or, in cases where the anticipated loads were small, on a microtensile stage (Deben Microtest, UK) with a 300 N load cell. In all cases, the cross-head speeds were maintained at $1\;\mathrm{mm}\;\min^{-1}$. In addition, we have used a nanoindentor (Hysitron Inc., USA) with *in situ* imaging function capable of applying indentation loads of the order of 1000 μN at selected locations on a material cross section.

Digital image correlation was used to monitor strains on samples during some of the tensile tests. The commercial software Vic-2D (Correlated Solutions, USA) was used for the measurement of displacements and strains on the sample surface. Further, an open source image processing software ImageJ (National Institute of Health, USA) was used to analyse micrographs obtained from the electron microscope, e.g. to determine the volume fraction of fibres on a cross section.

Results involving XRD were obtained using a powder X-ray diffractometer (Brucker AXS, USA) with a ${\mathrm{Cu}\;K}_{\alpha }$ source. Also, results involving electron microscopy were obtained from a field emission scanning electron microscope (FESEM, Sigma, Zeiss, Germany).

## 4. The stiffness of a bamboo culm

On a macroscopic scale, bamboo is a long tapered beam made of a radially graded transversely isotropic material. The grading in the radial direction results from the graded areal distribution of stiff fibre sheaths (around the vascular bundles) which are surrounded by relatively softer foam-like parenchyma cells. The fibres in the sheaths, in turn, have secondary cell walls that have a lignin and hemicellulose matrix reinforced by extremely stiff and largely crystalline cellulose microfibrils. In their study on Moso bamboo (*Phyllostachys pubescens*), Liu *et al.* [36] have explored the relationship between fibre area and tensile properties. While assessing the usefulness of bamboo as a structural material, we need to first determine exactly how this hierarchical material design strategy contributes to the overall stiffness. The investigation is complicated by the fact that the structure and properties of the different constituents at different scales are not known. However, we show that there exists a smaller set of key parameters which enable us to reliably predict the overall mechanical properties of a bamboo culm.

### 4.1. Predicting stiffness of the fibres

**Figure 9.**
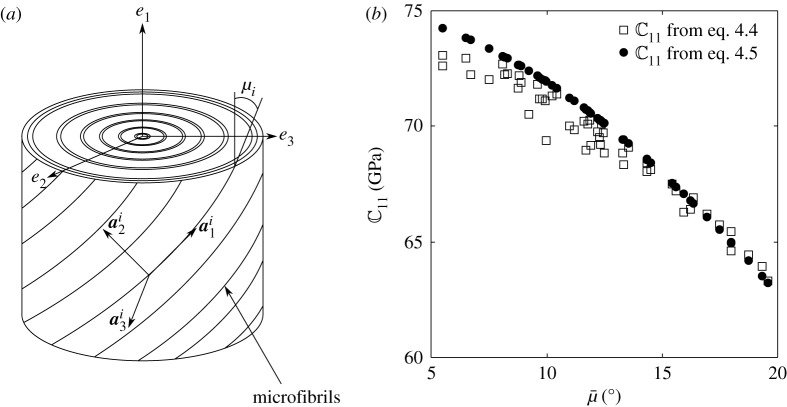
(*a*) Idealized multilamellar cell wall structure showing the axes $a_{\alpha }^{i}$ defining the orientations of the microfibrils. (*b*) Comparison of the values of the overall axial Young's modulus of a fibre calculated using equations (4.4) and (4.5).

We start by systematically calculating the properties of individual fibres. As discussed earlier, the fibre wall is composed of cellulose microfibrils oriented at a particular angle in a lamella. The number and thickness of the lamellae and the orientation of the microfibrils in each vary but some commonalities have been pointed out. Electron microscopic studies by Parameswaran & Liese [24] (see also [19, 37]) have revealed that there are generally *N* = 5–8 alternately thick and thin lamellae (thicknesses denoted by $t_{i}$) in the secondary cell wall, with the orientations being close to $\mu_{i}$ = 2–5${\;}^{\circ }$ and 85–90${\;}^{\circ }$ in the thick and thin layers, respectively. The somewhat idealized lamellar structure is shown in figure 9*a*. The orientation of the cellulose microfibrils defines an axis $a_{\alpha }^{i}$, whereas the longitudinal axis is denoted by $e_{1}$. The orientation angle of the microfibrils is $\mu_{i}=a_{1}^{i}\cdot e_{1}$.

#### 4.1.1. Fibre cell walls as fibre reinforced composites.

With the arrangement shown in figure 9*a*, each lamella, *i*, is transversely isotropic, with $a_{2}^{i}-a_{3}^{i}$ defining the plane of isotropy. The microfibril direction $a_{1}^{i}$ also coincides with the b axis of the cellulose crystal. The lignin and hemicellulose complex surrounding the microfibrils is considered to be isotropic. Estimates of the moduli of lignin and hemicellulose, obtained from experimental [38–40] and atomistic simulations [22], are in the range 2–6.7 and 3.5–8.4 GPa, respectively. Volume fractions of lignin and hemicellulose are 0.15 and 0.28, respectively [41]. Simple rule of mixtures applied to the lignin–hemicellulose complex suggests a modulus $E_{\mathrm{hl}}$ of about 7.8 GPa. The Poisson's ratio $\nu_{\mathrm{hl}}$ is taken to be 0.5, whereas the volume fraction $v_{\mathrm{hl}}=0.43$, so that the volume fraction of cellulose, $v_{c}=0.57$.

Hashin [42] has derived bounds on the stiffness of transversely isotropic composites reinforced with transversely isotropic fibres. For a transversely isotropic material, given the Young's moduli ${\bar{E}}_{i}^{A}$ and ${\bar{E}}_{i}^{T}$, shear moduli ${\bar{G}}_{i}^{A}$ and ${\bar{G}}_{i}^{T}$, Poisson's ratios ${\bar{\nu }}_{i}^{A}$ and ${\bar{\nu }}_{i}^{T}$, (where the superscripts *A* and *T* pertain to quantities in the axial and shear directions, respectively), and the transverse bulk modulus ${\bar{K}}_{i}$, the stiffness matrix ${\bar{C}}_{i}$ (connecting the stress σ to the strain ϵ) of the material of the *i*th lamella, in the $a_{\alpha }^{i}$ system is given as

4.1 $$\left\{\begin{array}{c} \sigma_{11}\\ \sigma_{22}\\ \sigma_{33}\\ \sigma_{12}\\ \sigma_{23}\\ \sigma_{23} \end{array}\right\}=\left(\begin{array}{cccccc} {\bar{E}}_{i}^{A}+4{\bar{K}}_{i}{\bar{\nu }}_{i}^{A} & 2{\bar{K}}_{i}{\bar{\nu }}_{i}^{A} & 2{\bar{K}}_{i}{\bar{\nu }}_{i}^{A} & 0 & 0 & 0\\ 2{\bar{K}}_{i}{\bar{\nu }}_{i}^{A} & {\bar{K}}_{i}+{\bar{G}}_{i}^{T} & {\bar{K}}_{i}-{\bar{G}}_{i}^{T} & 0 & 0 & 0\\ 2{\bar{K}}_{i}{\bar{\nu }}_{i}^{A} & {\bar{K}}_{i}-{\bar{G}}_{i}^{T} & {\bar{K}}_{i}+{\bar{G}}_{i}^{T} & 0 & 0 & 0\\ 0 & 0 & 0 & {\bar{G}}_{i}^{A} & 0 & 0\\ 0 & 0 & 0 & 0 & {\bar{G}}_{i}^{T} & 0\\ 0 & 0 & 0 & 0 & 0 & {\bar{G}}_{i}^{A} \end{array}\right)\left\{\begin{array}{c} \epsilon_{11}\\ \epsilon_{22}\\ \epsilon_{33}\\ 2\epsilon_{12}\\ 2\epsilon_{23}\\ 2\epsilon_{23} \end{array}\right\}.$$

The modulus of crystalline cellulose has been obtained by several authors through atomistic simulations [43, 44]. The unit cell of cellulose is not exactly symmetrical in the a and c directions (figure 5*c*) but the moduli in these directions turn out to be only marginally different, indicating that the cellulose microfibrils themselves can be approximated as transversely isotropic entities. We have performed atomistic simulations on a single crystalline periodic box consisting of cellulose $I_{\beta }$ (monoclinic form) unit cells, using the commercial software Materials Studio. The obtained values for the moduli in the axial b and transverse a or c directions are given by: $E_{c}^{A}=133$, $G_{c}^{T}=5.3$, $G_{c}^{A}=4.2$ and $K_{c}=14.7\;\mathrm{GPa}$, where subscript *c* denotes cellulose. The Poisson's ratio $\nu_{c}^{A}=0.087$. For the axial Young's modulus $E_{c}^{A}$, which is much higher compared with the other moduli, values reported in the literature are in the range of 124–155 GPa. In fact, crystalline cellulose microfibrils are comparable in stiffness to Kevlar.

The overall stiffnesses ${\bar{E}}_{i}^{A}$, ${\bar{K}}_{i}$ and ${\bar{G}}_{i}^{A}$ and axial Poisson's ratio ${\bar{\nu }}_{i}^{A}$ defining the stiffness matrix ${\bar{C}}_{i}$ of the *i*th lamella in the $a_{\alpha }$ system is given by Hashin [42]:

4.2 $$\begin{array}{rl} {\bar{K}}_{i} & =K_{\mathrm{hl}}+\frac{v_{c}}{1/(K_{c}-K_{\mathrm{hl}})-v_{\mathrm{hl}}/(K_{\mathrm{hl}}+G_{\mathrm{hl}})}\\ {\bar{E}}_{i}^{A} & =E_{c}^{A}v_{c}+E_{\mathrm{hl}}v_{\mathrm{hl}}+\frac{4{\left(\nu_{c}^{A}-\nu_{\mathrm{hl}}\right)}^{2}v_{\mathrm{hl}}v_{c}}{v_{c}/K_{\mathrm{hl}}+v_{\mathrm{hl}}/K_{c}+1/G_{\mathrm{hl}}}\\ {\bar{G}}_{i}^{A} & =G_{\mathrm{hl}}+\frac{v_{c}}{1/(G_{c}^{A}-G_{\mathrm{hl}})+v_{\mathrm{hl}}/2G_{\mathrm{hl}}}\\ \;\text{and}\;\;{\bar{\nu }}_{i}^{A} & =\nu_{c}^{A}v_{c}+\nu_{\mathrm{hl}}v_{\mathrm{hl}}+\frac{(\nu_{c}^{A}-\nu_{\mathrm{hl}})(1/K_{\mathrm{hl}}-1/K_{c})v_{c}v_{\mathrm{hl}}}{v_{c}/K_{\mathrm{hl}}+v_{\mathrm{hl}}/K_{c}+1/G_{\mathrm{hl}}}. \end{array}\}$$

For the properties of cellulose microfibrils (transversely isotropic) and lignin–hemicellulose complex (isotropic) chosen, the values of ${\bar{E}}_{i}^{A},\;{\bar{K}}_{i},\;{\bar{G}}_{i}^{A}$ and ${\bar{\nu }}_{i}^{A}$ are 74.3, 6.1, 2.1 and 0.19 GPa, respectively. Note that the axial Poisson's ratio and Young's modulus are well approximated by the rule of mixtures, because the last terms in equations (4.2)(b) and (d) are, in view of the large mismatch in stiffness between cellulose and the lignin–hemicellulose complex, small. Also, the transverse shear and Young's moduli and Poisson's ratios are given by bounds (also derived by [42]) and, according to the properties chosen,

4.3 $$2.0<{\bar{G}}_{i}^{T}<2.2\;\mathrm{GPa},\;6.0<{\bar{E}}_{i}^{T}<6.4\;\mathrm{GPa}\;\mathrm{and}\;0.46<{\bar{\nu }}_{i}^{T}<0.49.$$

To estimate the stiffness of the multilamellar secondary cell wall of the fibres in the $e_{\alpha }$ system, we take two heuristic approaches. Assume that the number of lamellae *N* ranges between four and eight. The thickness of lamella *i* is denoted by $t_{i}$ such that the total thickness is $T=\sum_{i=1}^{N}t_{i}$. It is known from microscopic studies on delignified fibres that the thick lamellae are about five times thicker than the thin ones (see [1, figure 46]). Total number *N* of alternate thick and thin lamellae with thickness ratio of 5 are generated with each lamella having a value of $\mu_{i}$. The orientations of the microfibrils $\mu_{i}$ are chosen from a uniform random distribution such that, in the thin lamellae $80^{\circ }<\mu_{i}<90^{\circ }$ and in the thick ones $0^{\circ }<\mu_{i}<10^{\circ }$.

In the first approach, stiffness of the multilamellar cell wall is then calculated using the simple rule of mixtures:

4.4 $$C=\sum_{i=1}^{N}\frac{t_{i}}{T}A_{i}^{-1}{\bar{C}}_{i}RA_{i}R^{-1},$$

where $A_{i}$ is the transformation matrix between $a_{\alpha }^{i}$ axis of the *i*th lamella and the global $e_{\alpha }$. R is the Reuter matrix, which is given by

$$R=\left(\begin{array}{cccccc} 1 & 0 & 0 & 0 & 0 & 0\\ 0 & 1 & 0 & 0 & 0 & 0\\ 0 & 0 & 1 & 0 & 0 & 0\\ 0 & 0 & 0 & 2 & 0 & 0\\ 0 & 0 & 0 & 0 & 2 & 0\\ 0 & 0 & 0 & 0 & 0 & 2 \end{array}\right).$$

Note that C denotes the effective stiffness matrix in the $e_{\alpha }$ system of the entire fibre wall containing four to eight lamellae.

In the second approach, the overall stiffness is calculated using

4.5 $$C={\bar{A}}^{-1}\bar{C}R\bar{A}R^{-1},$$

where $\bar{C}$ and $\bar{A}$ are calculated using the average orientation of the microfibrils,

4.6 $$\bar{\mu }=\sum_{i=1}^{N}\frac{t_{i}}{T}\mu_{i}.$$

The motivation for the second approach comes from the fact that the average microfibril orientation $\bar{\mu }$ is easily accessed through XRD or optical methods [32, 33, 45]. Indeed, the values of the component $C_{11}$ (the modulus in the axial direction) from several realizations performed with random values of *N* and $\mu_{i},i\in 1,N$, using equations (4.4) and (4.5) are compared in figure 9*b*. The average microfibril orientation, $\bar{\mu }$, has values between 5 and $20^{\circ }$ in our simulations (as also in reported experiments on bamboo, e.g. [46], and wood, e.g. [45]). The stiffness C calculated using $\bar{\mu }$ provides a reasonable bound to the overall modulus of the cell wall. In the subsequent discussions, unless otherwise mentioned, C for the cell wall will be assumed to be calculated using equation (4.5).

#### 4.1.2. The axial modulus through experiments.

The estimate for axial modulus obtained using equation (4.5) needs to be verified through experiments. Unfortunately, experimental determination of the mechanical properties of the cell wall is fraught with uncertainties (see [47] for a discussion of the issues involved). Here we have attempted two methods to verify if the estimates of stiffness that we determined theoretically in §4.1 are reasonable.

### 4.2. Tensile tests on fibre bundles

**Figure 10.**
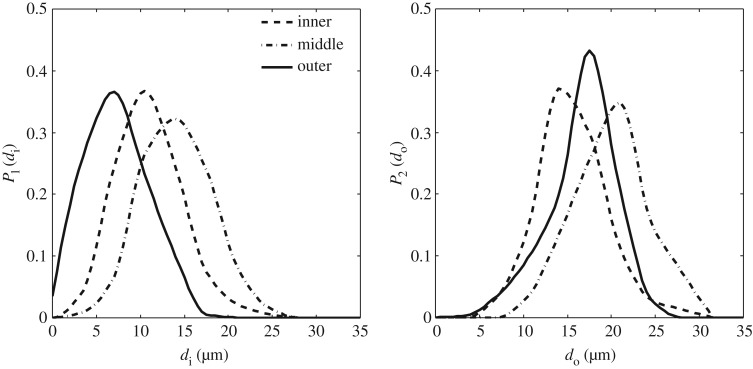
Experimentally determined probability density functions $P_{1}(d_{i})$ and $P_{2}(d_{o})$ at inner, middle and outer locations on a cross section.

The process of separating the fibres from parenchyma (detailed in §3) does not produce a single fibre but a bundle. A bundle contains hundreds of fibres and moreover, the shape of the cross section of the fibres depends on the fibre bundles drawn from location of the sheath from which the bundle is extracted. In fact, dimensions of several fibres extracted from inner, outer and middle locations on the cross section allow us to determine the probabilities $P_{1}(d_{i})$ and $P_{2}(d_{o})$ (shown in figure 10*a*,*b*) of finding a fibre with inner diameter $d_{i}$ and outer diameter $d_{o}$, respectively. Moreover, the fibres have different distributions of inner and outer diameters in the inner, outer and middle parts of the culm cross section; the fibres close to the outer region tend to have smaller inner diameters.

**Figure 11.**
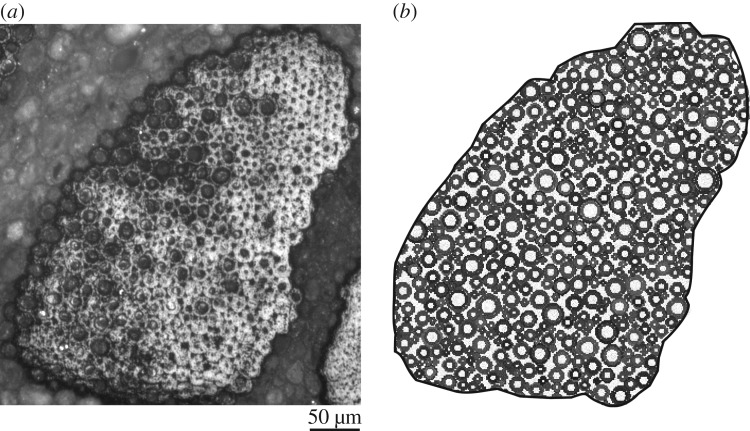
(*a*) Cross section of an extracted fibre bundle containing approximately 310 fibres. A synthetic bundle, containing 326 fibres, with $d_{i}$ and $d_{o}$ drawn from the distribution in figure 10 is shown in (*b*).

With the probability density functions at hand, using a standard algorithm for filling an arbitrary polygonal shape with circles, the irregular cross section of the bundle (e.g. the one shown in figure 11*a*) can be filled up with fibres having inner and outer diameters drawn from the distributions $P_{1}$ and $P_{2}$. This ensures that the synthetic bundle thus generated will have the same distribution of fibre cross sections as a real bundle. The algorithm for choosing a random numbers *x* (with a≤x≤b) such that they follow a given probability density p(x) is as follows (see [48, figure 7.3.2]):

— Generate a trial value $x_{\mathrm{try}}$ such that $x_{\mathrm{try}}=a+(b-a)\xi_{1}$, where $\xi_{1}$ is uniform random number between 0 and 1.
— Choose a function $f_{x}$ that has finite area and f(x)>p(x) for x≤x≤b.
— Determine $p(x_{\mathrm{try}})$ and $f(x_{\mathrm{try}})$ corresponding to $x_{\mathrm{try}}$.
— Generate another uniform random number $\xi_{2}$ between 0 and $f(x_{\mathrm{try}})$.
— Accept the trial value $x_{\mathrm{try}}$ only if $\xi_{2}\leq p(x_{\mathrm{try}})$.

The micrograph in figure 11*a* shows the entire cross section of an extracted fibre bundle, whereas figure 11*b* shows the corresponding synthetic bundle generated by filling up the cross-sectional shape with circles.

**Figure 12.**
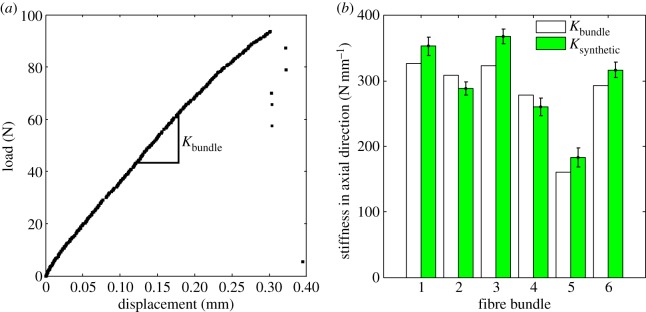
(*a*) Typical force–displacement response for tensile test on fibre bundle. Comparison between $K_{\mathrm{bundle}}$ and $K_{\mathrm{synthetic}}$ for six representative bundles drawn from different locations on the cross section is shown in (*b*).

Several extracted fibre bundles are tested on the microtensile set-up. The bundles of length 26±0.5 mm were gripped in the set-up with a gauge length L=10 mm. A typical force–displacement response is shown in figure 12*a*, from which, the slope $K_{\mathrm{bundle}}$ can be easily determined. Note that the bundle cross section has close-packed fibres bonded together with lignin, which is an order of magnitude more compliant compared with the cell wall material. Moreover, the volume fraction of the binder in the cross section is small. This suggests that we can safely neglect the contribution of the binder material on the axial stiffness of the bundle. The effective stiffness of a synthetic bundle is thus determined simply as

4.7 $$K_{\mathrm{synthetic}}=\frac{\sum_{k=1}^{M}A_{k}C_{11}({\bar{\mu }}_{k})}{L},$$

where *M* is the number of fibres in the bundle, $A_{k}$ the annular cross-sectional area of fibre *k* and ${\bar{\mu }}_{k}$ the average microfibril orientation angle (between 5 and $20^{\circ }$) randomly assigned to fibre *k* in the bundle. The axial modulus $C_{11}({\bar{\mu }}_{k})$ is obtained using equation (4.5).

The above model, at first glance, looks over-simplified. However, as shown in figure 12*b*, the stiffnesses $K_{\mathrm{bundle}}$ and $K_{\mathrm{synthetic}}$ are very close to each other for all the bundles tested. The fibre bundles in figure 12*b* are arranged such that samples 1 and 2 are from the inner part, 3 and 4 from middle and 5 and 6 are taken from the outer part. However, the diameter ranges (especially the range of the outer diameter) of the fibres do not show a strong distributional regularity. This is also evident from figure 10. Thus, the location of the fibre bundles does not seem to have a bearing on their stiffness as all bundles are constituted of fibres with almost identical distribution of inner and outer diameters. This result is remarkable given the fact that though a large number of factors are not known with certainty, knowledge of the average microfibril angle in the secondary cell walls, an estimate of the modulus of single crystalline cellulose and the size and shape distribution of the fibres in the cross section allows us to determine the axial modulus of a bamboo fibre quite closely. The radial variation in the axial modulus is primarily owing to the change in the fibre inner and outer diameter distribution.

### 4.3. Determination of stiffness through nano-indentation

Nano-indentation provides a quick and easy way of measuring mechanical properties of small volumes of materials *in situ*. Very low load, depth sensing indentation instruments are capable of making shallow indents as small as a few nanometres. The Young's modulus is typically measured during the recovery phase of the indentation, with the unloading process modelled as an elastic contact with a half space [49]. It has been shown by Oliver & Pharr [50] that the contact compliance is independent of the shape of the indenter and is given by:

4.8 $$C=\frac{dh}{dL}=\frac{\sqrt{\pi }}{2}\frac{1}{M_{r}}\frac{1}{\sqrt{A}},$$

where, the modulus $M_{r}$ is

4.9 $$\frac{1}{M_{r}}={\left(\frac{1-\nu^{2}}{E}\right)}_{\mathrm{indenter}}+{\left(\frac{1-\nu^{2}}{E}\right)}_{\mathrm{sample}},$$

for a substrate with isotropic properties. Here, *L* and *h* are the applied load and contact depth, whereas *A* is the projected contact area, obtained from the initial slope of the unloading curve [49]. For isotropic samples, this method of determining $E/(1-\nu^{2})$ for the substrate works well.

However, because the secondary cell walls of the fibres are anisotropic, the modulus $M_{r}$ measured by nano-indentation is not simply the modulus in the direction of indentation [51, 52]. The modulus $M_{r}$ will have to be computed as [51]:

4.10 $$\frac{1}{M_{r}}={\left(\frac{1-\nu^{2}}{E}\right)}_{\mathrm{indenter}}+16\pi^{2}{\left(\int_{0}^{2\pi }\alpha_{m}B_{km}^{-1}\alpha_{k}\;d\theta \right)}^{-1}.$$

**Figure 13.**
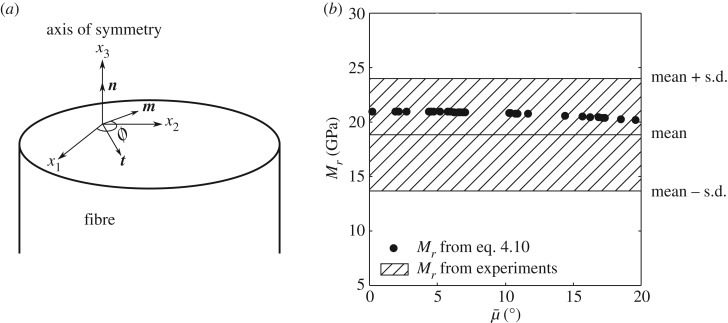
(*a*) The coordinate system and (m, n, t) vectors used to obtain estimates of the indentation modulus in equation (4.10). The variation of calculated indentation modulus $M_{r}$ with average microfibril orientation $\bar{\mu }$ is shown in (*b*). The indentation moduli obtained from nano-indentation experiments are superposed.

Barnett & Lothe [53] have derived the displacement Green's function for a point load applied on the boundary of an anisotropic half space. The tensor $B_{ij}$ arises in the Green's function and is defined according to the reference systems shown in figure 13*a*. The anisotropic material stiffness $C(\bar{\mu })$ is defined in the $x_{1},x_{2},x_{3}$ system, where, in our case, $x_{3}$ is along the fibre axis. The direction of indentation is normal to the surface, so that the direction cosines $\alpha_{i}$ are simply (0,0,1). The unit vector t lies on the surface, makes an angle *θ* with the $x_{1}$ axis and forms an orthonormal triad with m and n. Again, in the present case, n happens to be along $x_{3}$. Then, $B_{ij}$ is defined as [51],

4.11 $$B_{ij}(t)=\frac{1}{8\pi^{2}}\int_{0}^{2\pi }\{{\left(mm\right)}_{ij}-{\left(mn\right)}_{ik}{\left(nn\right)}_{km}^{-1}{\left(nm\right)}_{mj}\}\;d\phi ,$$

where *ϕ* is the angle between m and $x_{1}$. Here, we define, ${\left(ab\right)}_{ij}=a_{k}C_{ijkl}(\bar{\mu })b_{l}$. The term on the right-hand side of equation (4.10) can be evaluated numerically given the tensor $C(\bar{\mu })$. Comparison between the computed values and those obtained by measuring $M_{r}$ directly from nano-indentation is presented in figure 13*b*.

The value of $M_{r}$ calculated from equation (4.10) decreases with $\bar{\mu }$. When $\bar{\mu }$ is varied from 0 to $20^{\circ }$, $M_{r}$ ranges between 19 and 21 GPa. For experimentally determined values of $M_{r}$, the values of $\bar{\mu }$ at the point of indentation cannot obtained. However, the experimentally determined value of the modulus $M_{r}$ lies between 10 and 30 GPa. The mean value of the experimentally obtained indentation modulus, shown in figure 13*b*, is from 30 different nano-indentations on walls of different fibres in different cross-sectional samples from a culm. Values at mean ± standard deviation of the experimental data have also been shown to provide a measure of the scatter.

### 4.4. Properties of parenchyma

In order to obtain the modulus of the secondary cell walls of parenchyma cells, we followed a procedure similar to that described in §4.1.1 for fibres. Cellulose forms 33% of the parenchyma walls but the angle made by the cellulose microfibrils is much larger in parenchyma than in the fibres. The larger values of $\bar{\mu }$ makes parenchyma cell walls much more compliant than fibres. We estimated that the average microfibril orientation angle is 30–35${\;}^{\circ }$, which makes $M_{r}$ very low, ranging between 2 and 6 GPa only. The axial and indentation moduli for secondary cell walls of parenchyma and fibre cells are summarized in table 1 for the species of bamboo we tested. It can be expected that these values, dependent primarily on the average cellulose microfibril orientation, will also be good estimates for other species of bamboo.

**Table 1.** The axial and indentation moduli for parenchyma and fibre cell walls.

type of cell	axial modulus $C_{11}$ (GPa)	indentation modulus $M_{r}$ (GPa)
fibre	15–29	8–31
parenchyma	3–7	2–6

### 4.5. Overall axial properties of bamboo culm

**Figure 14.**
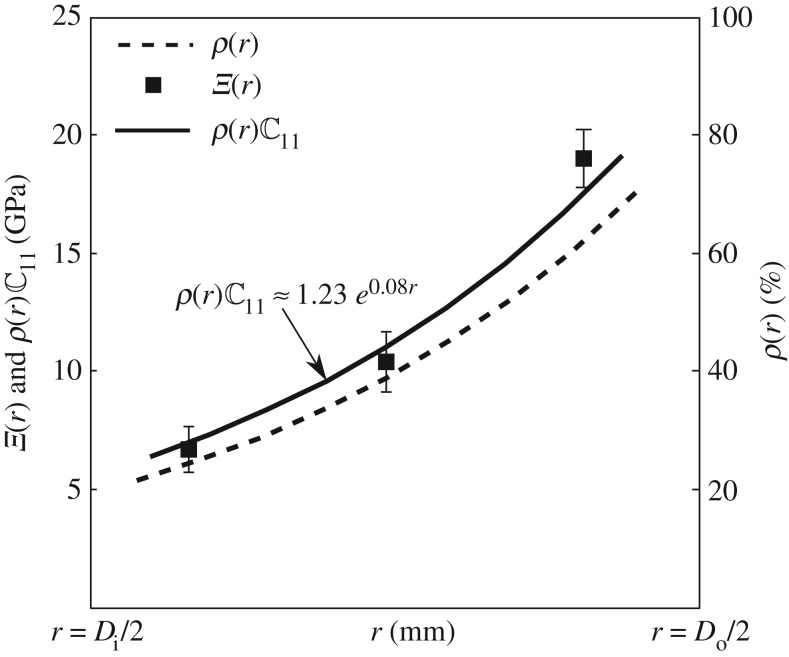
The variation with radius *r* of the areal density ρ(r) of fibre clearly follows the variation in $\rho (r)C_{11}$, which, in turn, is close to the experimentally obtained axial moduli Ξ(r) shown by solid squares. (Note: $D_{i}$ and $D_{o}$ are inner and outer diameter of bamboo culm, respectively.)

Radial variation ρ(r) of areal density of fibres on the cross section of a culm is shown by dotted lines in figure 14. As also shown by the micrographs in figure 3, the areal density increases sharply towards the outer part of the culm. Tensile tests have been conducted on several 3 mm thick specimens with their gauge lengths aligned in the longitudinal direction (figure 8). The modulus Ξ(r) obtained from these tests is also plotted on figure 14. Clearly, the overall axial modulus of the culm follows the variation of the areal density ρ(r) very closely. This implies that the overall modulus, at least in the axial direction, relies entirely on the stiffness of the fibres.

Indeed, as shown in figure 14, if we calculate the overall modulus as $\rho (r)C_{11}$, where $C_{11}$ is calculated using equation (4.5), it matches quite closely with what is measured from the tensile tests. The overall axial modulus does not depend significantly on the stiffness of the parenchyma cells.

These results can be compared with those of Habibi *et al.* [4] and Dixon & Gibson [5] obtained on other species of bamboo. In both these studies, the modulus was seen to depend on the distribution of fibres. In the latter case, Moso bamboo exhibited an average stiffness of about 15 GPa, but varied very little over the thickness. Habibi *et al.* [4] report a variation in stiffness from 10 to 16 GPa. Although the values of stiffness are comparable to the species characterized by us, we detect a stronger grading of the stiffness. Moreover, we also trace the origin of the stiffness to the stiffness of single crystal cellulose and the characteristic microfibril angle.

Overall axial modulus, in the final analysis, turns out to be deceptively simple to determine. We need to know the range of average microfibril orientation angles in the fibres and the areal distribution of the fibres over the cross section. Thus, ρ(r) and $\bar{\mu }$ are the most important parameters that dictate the stiffness of bamboo and determining these hold the key to predicting the axial stiffness. Once determined (we have addressed the determination of the mean microfibril orientation in bamboo [46], and it has been determined for varied types of wood through different techniques [32, 33, 45]), assuming that the microfibrils are single crystalline cellulose $I_{\beta }$, the overall axial modulus can be calculated in a straightforward manner. The hemicellulose–lignin complex surrounding the cellulose in the microfibrils and the foam-like parenchyma around the fibre bundles are too soft to play any significant role in determining stiffness. Note that this does in no way rule out the fact that the softer phases may have important functions in determining the toughness and arresting propagating cracks.

Thus, the culm turns out to be a naturally functionally graded material with axial stiffness varying as $\Xi_{0}e^{\beta r}$, with $\Xi_{0}=1.23\;\mathrm{GPa}$ and $\beta ≃0.08\;{\mathrm{mm}}^{-1}$.

## 5. Conclusion

We have studied the morphology and stiffness of a local variety of bamboo with a view to establish an implementable method of linking the former to the latter. We have shown that

1. The axial modulus of a bamboo internode can be estimated if we have reliable measures of (i) the average microfibril angle in the secondary cell walls of the sclerenchymatous fibres, (ii) areal distribution of the fibres, and (iii) size distribution of the fibres.
2. The estimates of stiffness obtained using simple ideas borrowed from the mechanics of composite materials have been tested against experiments both on bundles of fibres and samples drawn from the culm.
3. Bamboo is a radially graded, transversely isotropic composite material. For the species studied, axial modulus varies with radius as $1.23e^{0.08r}\;\mathrm{GPa}$.

It should be noted that from a structural point of view, the various aspects of the microstructure are poorly understood. These include the role and exact properties of the soft foam-like parenchyma and hemicellulose–lignin complex surrounding the cellulose microfibrils in the sclerenchyma walls. While our study has shown that the axial stiffness is not strongly affected by our lack of knowledge in these aspects, their role in arresting fracture cannot be ruled out. This will be discussed in a forthcoming paper.

## Footnotes

1 Both triclinic (*α*) or monoclinic (*β*) unit cells are possible, though the *β* structure is abundant in bamboo.

## Data accessibility

Supporting data for this study can be accessed via the Dryad Digital Repository http://dx.doi.org/10.5061/dryad.5ch51 [54]. The supporting data contains (i) micrographs of bamboo microstructure other than those mentioned in the manuscript; (ii) immunofluorescence images of bamboo microstructure other than those mentioned in the manuscript (Initial−IC_bamboo_1406.pptx); (iii) data of XRD study and the calculations thereof (degree of crystallinity.xlsx); (iv) data used for generating probability density functions (ID_OD_Size_Distribution.xlsx); and (v) Matlab script for filling a circle (for_CIRC_shape.m) and an arbitrary-shaped polygon (for_any_shape.m) with circles.
